# 

**Published:** 1870-03

**Authors:** 


### CONTENTS:

ORIGINAL COMMUNICATIONS:

ART. I. Cases of Trichiniasis, and Successful Treatment. By C. B. Reed, M.D., - - - 130
" II. Reply to a Criticism of Z. C. McElroy, M.D. By H. Culbertson, M.D., - - - 134
" III. Address before the Alumni Association of Rush Medical College, Feb. 2, 1870. By A. E. Ames, M.D., - - 145
" IV. Aortic Aneurism. Pathological Specimen. By F. J. VanVorhis, M.D., - - - 155

ORIGINAL TRANSLATIONS:

ART. I. Analysis of a Case of imperfect Bisexual Hermaphrodism in Man. By Dr. E. Goujon of Paris, - - - 156

EDITOR'S BOOK TABLE, - - - - - - - 173

EDITORIAL:

Rush Medical College, - - - - - - - 175
Coroner's Inquests, - - - - - - - 175
News Items, - - - - - - - - - - 176

LOOT.

On Earth Closets, - - - - - - - - 178
Hydrate of Chloral, - - - - - - - 183
Partial Paraplegia, - - - - - - - 186
Paste, - - - - - - - - - - - 187
Morphine vs. Atropine, - - - - - 187
Kerosene, - - - - - - - - - - 187
Alcohol on Sponge, - . - - - - - 187
The Perils of Fashion, - - - - - - 188
Dissection Wounds, - - - . - - - 189
Remarkable Operation. Extirpation of a Kidney, - - - - - - - - - 189
Water in Alcohol, - - - - - - - - 190
Bromide of Potassium for the Troubles of Teething, - - - - - - - - - 190

POETRY, - - - - - - - - - - - - - 191

